# Linoleic acid binds to SARS-CoV-2 RdRp and represses replication of seasonal human coronavirus OC43

**DOI:** 10.1038/s41598-022-23880-9

**Published:** 2022-11-09

**Authors:** Anna Goc, Waldemar Sumera, Matthias Rath, Aleksandra Niedzwiecki

**Affiliations:** grid.418580.0 Dr. Rath Research Institute, 5941 Optical Ct., San Jose, CA 95138 USA

**Keywords:** Microbiology, Virology

## Abstract

Fatty acids belong to a group of compounds already acknowledged for their broad antiviral efficacy. However, little is yet known about their effect on replication of human coronaviruses. To shed light on this subject, we first screened 15 fatty acids, three lipid-soluble vitamins, and cholesterol, on SARS-CoV-2 RdRp, and identified the four fatty acids with the highest RdRp inhibitory potential. Among them, linoleic acid was found to have the greatest interaction with SARS-CoV-2 RdRp, with its direct binding to the cavity formed by the RNA double helix and protein. Linoleic acid forms hydrophobic interactions with multiple residues, and at the same time forms electrostatic interactions including the hydrogen bond with Lys593 and Asp865. In line with these results, a dose-dependent inhibition of HCoV-OC43 replication in vitro was observed, additionally strengthened by data from in vivo study, which also confirmed anti-inflammatory potential of linoleic acid. Based on these results, we concluded that our study provides a new understanding of the antiviral properties of fatty acids against human coronaviruses including the SARS-CoV-2 strain. Particularly, they lays down a new prospect for linoleic acid’s RdRp-inhibitory activity, as a candidate for further studies, which are warranted to corroborate the results presented here.

## Introduction

The SARS-CoV-2 strain (earlier described as 2019-nCov, 2019-CoV-2, and nCoV-2019) has been identified as a causative agent responsible for the outbreak of pneumonia in Wuhan, China, in 2019 and the subsequent COVID-19 pandemic. Since then, several other variants have been identified, such as: Alpha (B.1.1.7), Beta (B.1.351), Gamma (P.1), Delta (B.1.617.2), Epsilon (B.1.429/B.1.427), Zeta (P.2), Eta (B.1.525), Theta (P.3), Iota (B.1.526), Kappa (B.1.617.1), and Lambda (C.37), as well as the more-recently reported Mu (B.1.621) and Omicron (B.1.1.529)^1–3^. There are four widespread human coronaviruses (i.e., HCoV-229E, HCoV-NL63, HCoV-OC43, and HCoV-HKU1) known to cause rather mild respiratory infection, in contrast to three others (i.e., SARS-CoV-1, identified in 2003; MERS-CoV, identified in 2013; and SARS-CoV-2, identified in 2019) that cause severe lung dysfunction^4,5^.

SARS-CoV-2 is a RNA type virus that contains a positive, single-stranded and polycistronic RNA (+ ssRNA), enveloped with four structural proteins, i.e., the spike (S) protein, envelope (E) protein, membrane (M) protein, and the nucleocapsid (N) protein^6–9^. In addition to these four structural proteins, its ~ 30 kb genomic RNA (gRNA) encodes 16 non-structural and auxiliary proteins (nsps), including RNA-dependent RNA polymerase (RdRp), 3-chymotrypsin-like protease (3CLpro), papain-like protease (PLpro), and helicase. After entry into the host cell, the SARS-CoV-2 gRNA, which has 14 open reading frames (ORFs), undergoes transcription and translation, resulting in the multiplication of viral virions. ORFs 1a and 1b encode two replicase polyproteins (PP1a and PP1ab), which are cleaved by PLpro and 3CLpro, generating nsps. Two proteins, nsp12 (i.e., RdRp) and nsp13 (i.e., helicase), are involved in guiding the viral genome and protein synthesis, with the assistance of nsp7 and nsp8a/nasp8b as cofactors^6,7^. Therefore, nsp12 is considered a primary target for antiviral inhibitors, i.e., synthetic agents such as nucleotide analogs, and naturally occurring compounds, e.g., polyphenols^10–14^. ORFs 2–14 encode four viral structural proteins and nine auxiliary factors, which participate in the viral capsid formation.

In coronaviruses, RdRp catalyzes the synthesis of their own RNA by using the (+)RNA strand as a template to produce a complementary (−)RNA strand, starting from a 3′‐poly‐A tail. Two possible mechanisms for gRNA synthesis by RdRp are recognized as de novo (primer‐independent) and primer‐dependent RNA synthesis^15–17^. Upon primer‐independent synthesis, gRNA is progressively synthesized through the formation of a phosphodiester bond composed of a 3′‐hydroxyl group bond of a first nucleotide and the 5′‐phosphate group of the second one. Upon primer‐dependent synthesis, an RNA-complementary strain is synthetized using the template and created by an oligonucleotide-prime-guided base pairing.

As mentioned above, the replication complex of SARS-CoV-2 is composed of a catalytic subunit, nsp12, as well as two accessory subunits, nsp8a/nsp8b and nsp7. The nsp12 subunit contains an N-terminal nidovirus RdRp-associated nucleotidyltransferase (NiRAN) domain, an interface domain, and a C-terminal RdRp domain. The C-terminal RdRp domain, formed by the conserved polymerase motifs A to G, comprises the three domains named as “fingers”, “palm” and “thumb”. Nsp7 and nsp8b bind to the “thumb” subdomain, whereas an additional copy—nsp8a—binds to the “fingers” subdomain. The core structure of RdRp that participates in RNA synthesis, i.e., the active site of RdRp, is surrounded by these three subdomains, and is located in the “palm” domain, which contains a highly conserved architecture of α‐helices, antiparallel β‐strands, RNA-recognition motif, and catalytic aspartates. The active site of RdRp is configured similarly to RNA polymerases of other RNA viruses, including retroviruses, and is quite easily reachable, making it a therapeutic target of interest^14–17^.

Fatty acids (FAs) belong to the large group of bioactive compounds of either plant or animal derivation^18,19^. They are amphiphilic in nature since they comprise a lipophilic short, medium or long fatty acid chain, either saturated or unsaturated, and the hydrophilic “head”. Their health benefits have been observed in various aspects of cardiovascular diseases, as well as neurological, metabolic and immunological disorders^20–23^. This is because FAs, and especially essential fatty acids (EFAs), are heavily involved in shaping, controlling, and determining the course of the vast majority of physiological and biochemical processes in humans and animals. Thus, they have a meaningful clinical impact when their availability in diet or metabolism is altered^18–24^.

Deficiencies of EFAs such as cis-linoleic acid (LA, 18:2, n-6) in the human diet are uncommon, owing to their abundant presence in widely available and inexpensive agricultural products^21,23,25^. Presently, LA comprises 7% of daily calories consumed by Western populations, despite exhortations to shift consumption from favoring n-6 regardless of the shift in consumption from favoring n-6 to n-3 polyunsaturated acids (PUFAs). The intake of LA’s metabolic derivatives, i.e., arachidonic acid (AA, 20:4, n-6) and gamma-linolenic acid (GLA, 18:3, n-6), as well as alpha-linolenic acid (ALA, 18:3, n-3) and its derivatives, eicosapentaenoic acid (EPA, 20:5, n-3) and docosahexaenoic acid (DHA, 22:6, n-3), continues to be largely unchanged^25,26^. Although LA has been considered non-functional for the brain because of its low tissue contents (< 2% of total FAs), it is nevertheless a substrate for AA, a progenitor of pro-inflammatory eicosanoids and ALA, a precursor for anti-inflammatory eicosanoids. Subsequent metabolites of these eicosanoids may even function as anti-hypertensive and anti-atherosclerotic compounds^27–29^.

Here, we examined several important FAs, lipid-soluble vitamins, and cholesterol for their potential in inhibiting of SARS-CoV-2 RdRp. We were able to show that LA, AA, EPA, and ALA, which belong to the PUFA series, have a substantial inhibitory efficacy. Among them, LA exhibited the highest binding affinity to RdRp protein, and concurrent experiments using replication-competent rVSVΔG-SARS-CoV-2 particles revealed that LA impedes its replication in vitro. This was corroborated by an in vivo study, the results of which revealed lower viral load in the mouse lungs after intratracheal and oral administration of LA. In conclusion, this study documents that PUFAs, in particular LA, by acting directly on RdRp, could decrease viral replication. Although further study is warranted, the identification of this compound as a prospective antiviral agent forms the basis of further scientific investigations.

## Results

### Evaluation of inhibitory properties of FAs, lipid-soluble vitamins, and cholesterol on activity of SARS-CoV-2 RdRp

**Figure 1:**
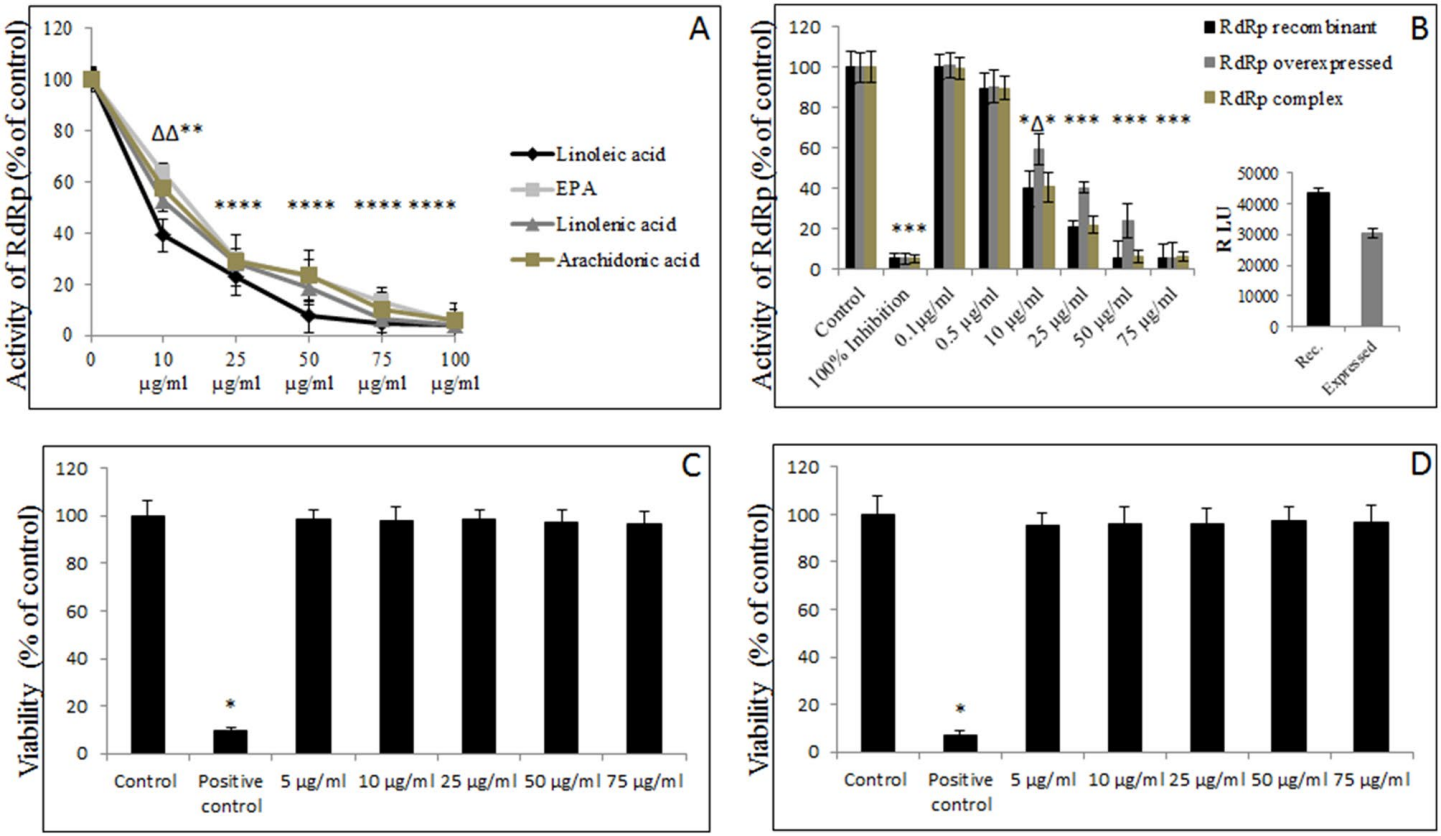
Effects of PUFAs on activity of SARS-CoV-2 RdRp. (**A**) Recombinant RdRp was incubated with selected FAs at designated concentrations for 15 min at RT. Mix composed of NTPs and RNA template was then added, and reaction was carried out for 2 h at 34 °C. Signal was measured after 10 min at extension/emission = 488/535 nm with spectrofluorometer. Data are presented as % of control – 1.0% DMSO. (**B**) Recombinant RdRp or its mixture with accessory nsp7/nsp8 and RdRp overexpressed in VeroE6 cells was incubated with designated concentrations of LA for 15 min at RT. Mix composed of NTPs and RNA template was then added, and reaction was carried out for 2 h at 34 °C. Signal was measured after 10 min at extension/emission = 488/535 nm with spectrofluorometer. Data are presented as % of control – 1.0% DMSO, 100% inhibition – RdRp that was boiled at 100 °C and additionally exposed to UV for 30 min; insert – RFU readouts as an expression of activity of RdRp recombinant versus RdRp overexpressed. (**C**) Viability of cells treated only with designated concentrations of LA determined by measuring of absorbance after 24 h. (**D**) Viability of cells after treatment with designated concentrations of LA in the presence of viral particles determined by absorbance readouts after 24 h, EPA – eicosapentaenoic acid, LA – linoleic acid; ∆ *p* ≤ 0.01, * *p* ≤ 0.001.

We tested 15 FAs, three lipid-soluble vitamins, and cholesterol, for their ability to inhibit activity of SARS-CoV-2 RdRp. As presented in Table 1, PUFAs revealed the highest inhibitory effect at 1.0 mg/ml concentration, while saturated fatty acids were the least effective. Out of five tested PUFAs, the LA showed the highest dose-dependent inhibitory effect with 57% inhibition of recombinant RdRp observed at 10.0 μg/ml (Fig. 1A,B). Using the mixture of nsp12 and its accessory factors nsp7 and nsp8, a dose-dependent inhibitory effect with 56% inhibition of recombinant RdRp observed at 10.0 μg/ml. When lysates of cells overexpressing RdRp were used, the dose-dependent inhibitory effect sustained with 42% inhibition of RdRp observed at 10.0 μg/ml (Fig. 1B). We verified that the viability of cells exposed to LA alone or together with the viral particles, was not affected (Fig. 1C,D).

**Table 1:** Effect of lipids on activity of SARS-CoV-2 RdRp.

Tested fatty acids (1.0 mg/ml)	Inhibition of RdRp activity (% ± SD)
**Polyunsaturated**	
Arachidonic acid	100 ± 0.02*
Docosahexaenoic acid	52.4 ± 3.7^^^
Eicosapentaenoic acid	100 ± 0.04*
Linoleic acid	100* ± 0.05*
Linolenic acid	100 ± 0.03*
**Monounsaturated**	
Palmitoleic acid	96.3 ± 1.9^^^
Petroselinic acid	97.7 ± 2.1^^^
Oleic acid	98.9 ± 1.2^^^
Erucic acid	75.9.1 ± 5.8^^^
**Saturated**	
Arachidic acid	
Caprylic acid	18.6 ± 2.1
Myristic acid	19.3 ± 3.1
Palmitic acid	13.2 ± 3.9
Stearic acid	15.9 ± 2.9
Undecenoic acid	16.3 ± 3.1
**Lipid-soluble vitamins and others**	
Vitamin D3 (1,25-dihydroxycholecalciferol)	36.9 ± 6.3^#^
Vitamin E (alpha-tocopherol)	39.2 ± 4.2^#^
Vitamin A (retinol)	65.3 ± 3.9^^^
Cholesterol	46.3 ± 4.1^#^

1.0 mg/ml of tested FAs, lipid-soluble vitamins, and cholesterol were first incubated with the mix containing SARS-CoV-2 RdRp for 20 min at RT and then supplemented with NTPs and template, and further incubated for an additional 2 h at 34 °C. Plates were then developed with 1 × fluorescence dye for up to 10 min. Fluorescence was measured at Ex/Em = 480/535 nm; # *p* ≤ 0.05, ^*p* ≤ 0.01, *p ≤ 0.001.

### Binding of linoleic acid to RdRp of SARS-CoV-2

**Figure 2:**
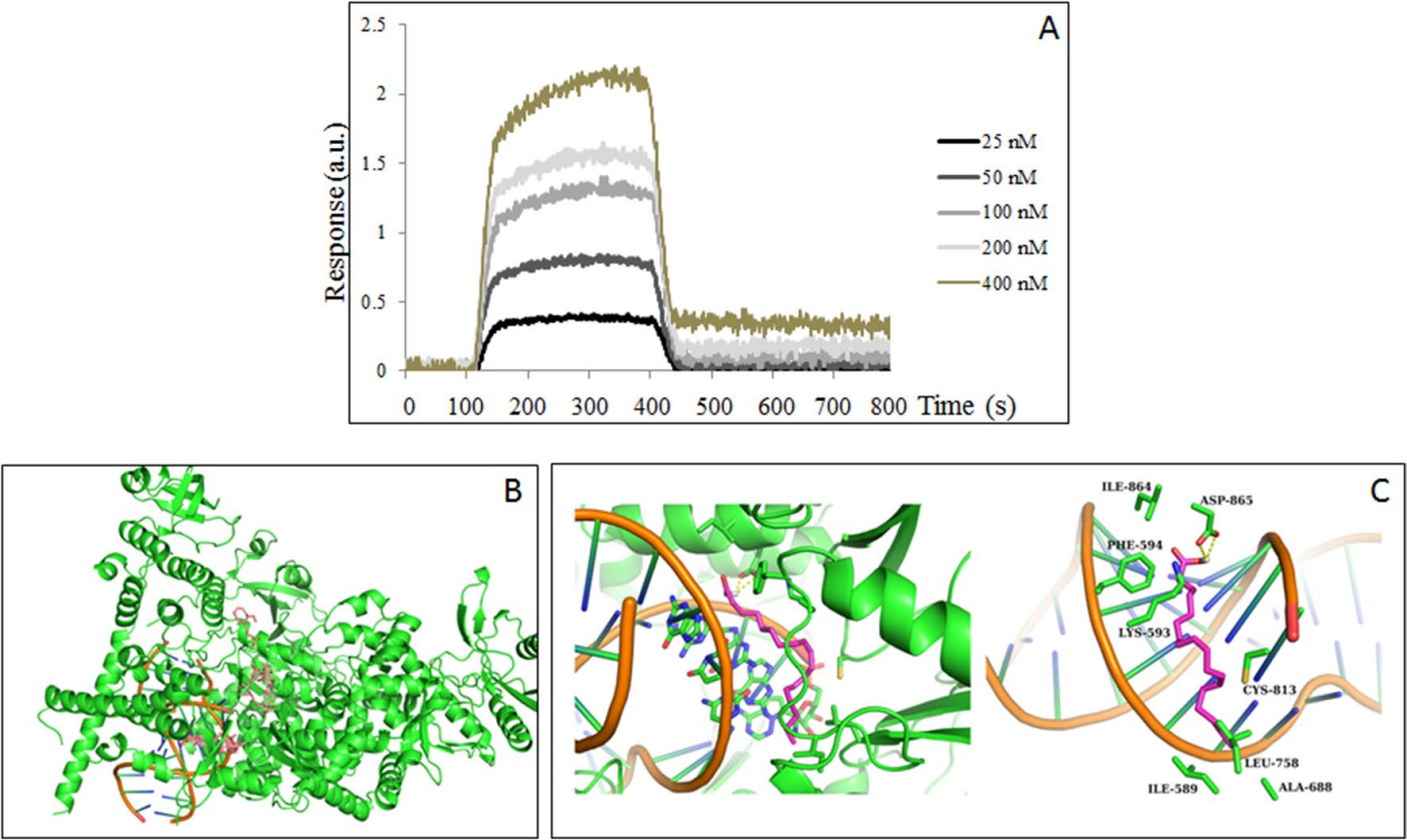
Binding of LA to SARS-CoV-2 RdRp. (**A**) Evaluation of the binding affinity and affinity parameters of LA to RdRp by SPR. The equilibrium dissociation constant (K_D_ Value) with ka and kd were calculated. (**B**) Binding modes distribution of LA. The protein and RNA are represented by the green and orange cartoon models, respectively, and the 9 binding modes were represented by the pink stick model. (**C**) Best binding mode of LA. Protein and RNA are shown in green and orange ribbon models, and small molecules were shown in purple stick models (C in purple and O in red). The residues within 4 Å nearby LA are depicted in green stick models (C in green and O in red), and hydrogen bonds were shown by yellow dashed lines, LA- linoleic acid.

To obtain deeper understanding of the nature of LA and RdRp interaction, we utilized SPR assay that allows determining the binding affinity parameters. Select protein-grafted regions in the SPR images were analyzed, and the average reflectivity variations of the chosen areas were plotted as a function of time. Real-time binding signals were recorded and analyzed by Data Analysis Module (DAM) (Plexera Bioscience, Seattle, WA). Kinetic analysis was performed and revealed that the equilibrium dissociation constant (K_D_ value) was 9.30 × 10^–7^ M with ka = 1.57 × 10^3^ M^−1^·s^−1^ and kd = 1.46 × 10^−3^ s^−1^ (Fig. 2A).

Next, we evaluated the binding characteristic of LA to the RdRp of SARS-CoV-2. The scores of 9 binding modes of linoleic acid with target protein RdRp obtained by molecular docking were generated as shown in Table 2. The Vina docking score was based on the experimental binding free energy value as the fitting object. The scores of the 9 binding modes obtained were between -7.0 and -4.5 kcal/mol, and the best binding mode was -7.0 kcal/mol. The free energy of binding and K_D_ value could be converted according to formula 1 [Δ DG = − RT * In(Kd)] and R = 8.314 J·mol^−1^·K^−1^; T (K) = 298.15 K (25 °C); 1 kcal = 4185.851820846 J. According to the experimental K_D_ value of 9.30 × 10^–7^ M, the binding free energy was calculated as − 8.22 kcal/mol. The binding modes obtained by molecular docking were distributed near the RNA binding pocket, in which the best binding location was between the RNA and the protein (Fig. 2B,C). In the best binding mode, LA was bound to the cavity formed between the RNA double helix and the protein. One of its sides remains in contact with the RNA base pair, and the other side forms a hydrophobic interaction, and hydrogen bonds with the amino acid residues on the protein. The long alkyl chain of the LA molecule forms hydrophobic interactions with Ile589, Leu758, Ala688, Cys813, Lys593, Phe594, Ile864, and the terminal carboxyl group forms electrostatic interactions with Lys593. The hydroxyl group forms a hydrogen bond with Asp865.

**Table 2:** Docking scores for 9 binding modes obtained by molecular docking.

Models	Docking score (kcal/mol)
Model1	− 7.0
Model2	− 6.6
Model3	− 6.4
Model4	− 5.9
Model5	− 5.5
Model6	− 5.2
Model7	− 4.9
Model8	− 4.8
Model9	− 4.8

### Effect of linoleic acid on HCoV-OC43 replication *in vitr*o

**Figure 3:**
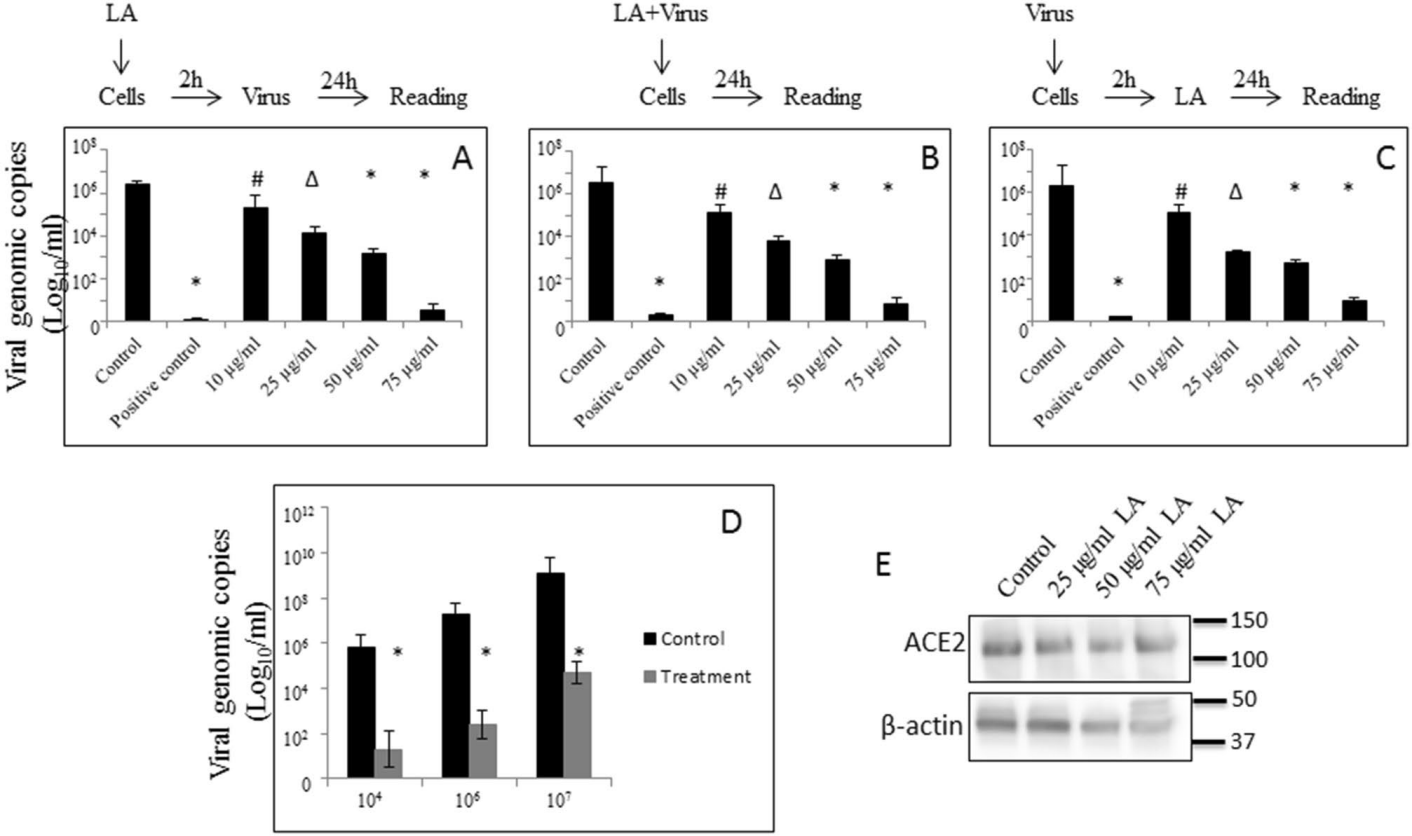
Effects of LA against viral replication in vitro. (**A**) Pre-treatment effect of increasing concentrations of LA on HCoV-OC43 replication. (**B**) Concurrent effect of increasing concentrations of LA on HCoV-OC43 replication. (**C**) Post-treatment effect of increasing concentrations of LA on HCoV-OC43 replication. (**D**) Effect of concurrent application of single 75 µg/ml concentration of LA and different concentrations of HCoV-OC43 virus. (**E**) Effect of increasing concentrations of LA on ACE2 expression at protein level. The effect against RdRp activity was determined by measurement of viral particles in conditioning media of MRC-5 cells by RT-qPCR and represented as viral copies/ml. The effect on ACE2 expression was determined in A549 cells by Western blot. Data are presented as mean + / − SD; control – cells exposed to virus and 0.05% DMSO, positive control – 100% dead cells, i.e., cells that were exposed to 100% of DMSO and additionally to UV for 30 min, *LA* linoleic acid; # *p* ≤ 0.05, ∆ *p* ≤ 0.01, * *p* ≤ 0.001.

To gain more information about the effects of LA on activity of RdRp in vitro, we arranged 3 experimental treatment patterns of MRC-5 cells with LA: (a) pre-incubation of cells with LA for 2 h before exposure of cells to HCoV-OC43, respectively (b) simultaneous addition of LA and HCoV-OC43, and (c) addition of LA 3 h after exposure of cells to HCoV-OC43, respectively. In all these 3 experimental configurations, we observed a dose-dependent decrease of viral particles in conditioning media of cells ranging from 1.1 to 3.6 log_10_ at LA concentrations from 10.0 μg/ml to 50 μg/ml. In more detail, the LA pre-treatment experiment revealed 1.1–3.2 log_10_ reduction in viral load, at LA concentrations from 10.0 to 50 μg/ml. Simultaneous addition of LA caused 1.3–3.6 log_10_ reduction in viral load, at LA concentrations from 10.0 to 50 μg/ml. The LA post-treatment experiment revealed 1.2–3.6 log_10_ reduction in viral load. In all three experimental designs, viral particles were undetectable in samples treated with 75 μg/ml of LA (Fig. 3A–C). Additionally, with the highest concentration of LA (i.e., 75 μg/ml) and different doses of HCoV-OC43 applied simultaneously, significant inhibition of viral replication was observed. As shown in Fig. 3D, 4.5-fold reduction in viral genomic copies was achieved at 10^4^ of the initial viral application, 4.8-fold at 10^6^ of the initial viral application, and 4.4-fold at 10^7^ of the initial viral application. Also, no changes in ACE2 expression at the protein level was observed upon 24 h treatment with concentrations of LA ranging from 25 to 75 µg/ml (Fig. 3E, and Supplementary Fig. S1).

### Effect of linoleic acid on viral replication in vivo

**Figure 4:**
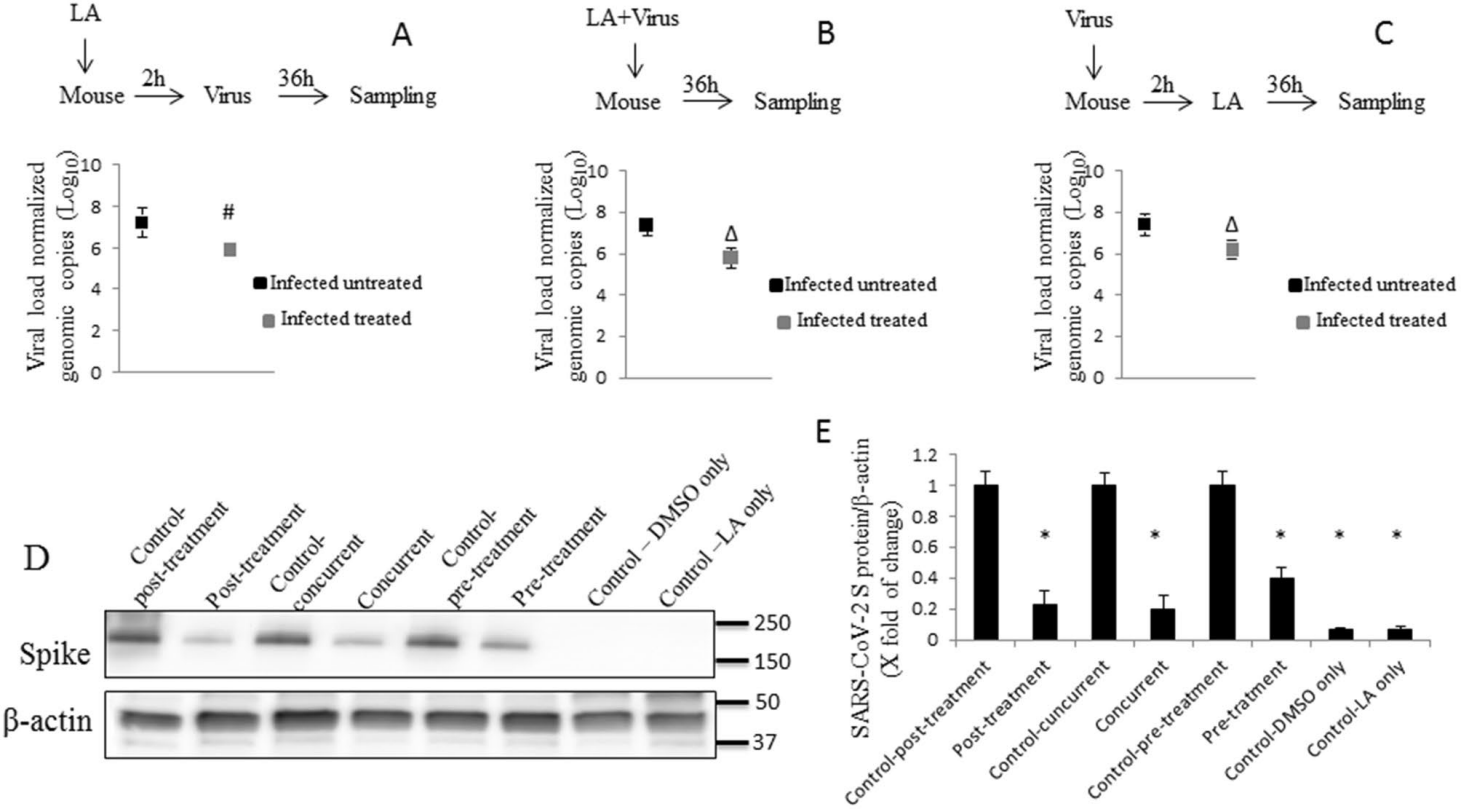
Effect of intra-tracheal administration of LA against viral replication in vivo. (**A**) Pre-treatment effect of LA on HCoV-OC43 replication in lung tissue of K18-hACE2 C57BL/6 J mice. (**B**) Concurrent effect of LA on HCoV-OC43 replication in lung tissue of K18-hACE2 C57BL/6 J mice. (**C**) Post-treatment effect of LA on HCoV-OC43 replication in lung tissue of K18-hACE2 C57BL/6 J mice. (**D**) SARS-CoV-2 spike protein detection in mice lungs with western blot. (**E**) Quantification of SARS-CoV-2 spike protein performed as described in Material and Method section. Data are presented as mean + / − SD; uninfected untreated animal n = 3, uninfected treated with LA animal n = 3, infected untreated animal n = 8, infected treated animal n = 8; viral burden in lung tissue was determined by RT-qPCR and represented as viral copies normalized to mouse Pol2Ra; LA – linoleic acid; # *p* ≤ 0.05, ∆ *p* ≤ 0.01, * *p* ≤ 0.001.

In order to verify whether or not LA can inhibit viral burden in lung tissue of animals as well, we used K18-hACE2 C57BL/6 J mice that were randomly divided into 8 experimental groups (Table 3). The mice were infected intratracheally (non-surgically) with a 1 × 10^5^ infectious dose of HCoV-OC43 or a 3 × 10^5^ infectious dose of rVSVΔG-SARS-CoV-2-S-D614Gd21-NLucP. LA was administrated either intratracheally or via oral gavage. Intratracheal administration of LA was further conducted in three schemes (Fig. 4A–C). Using TaqMan principle, as a measure of viral burden in the lungs, showed a significant 1.10 log reduction in pre-treated group, 1.5 log_10_ in concurrent group and 1.2 log_10_ post-treated group, compared with their representative untreated infected controls. Western blot for HCoV-OC43 spike protein confirmed its lower presence in the lungs of treated infected animals compared with the untreated infected mice (Fig. 4D). Quantification of the SARS-CoV-2 spike protein revealed ~ 1.5 × higher density of spike protein corresponding band in the infected untreated animals, compared with animals infected and pre-treated with LA, as well as ~ 4.0 × higher density of spike protein corresponding bands in the infected untreated animals compared with the animals treated concurrently with LA, and the post-treated mice (Fig. 4E, and Supplementary Fig. S2).

**Table 3:** Experimental animal groups of in vivo study.

Group	Treatment
1	Uninfected animals intratracheally injected with 0.5% DMSO
2	Intratracheally infected animals with intratracheally injected 0.5% DMSO
3	Uninfected animals intratracheally injected with 75 μg LA
4	Intratracheally infected animals and intratracheally injected with 75 μg LA
5	Uninfected animals gavaged with 5.0% ethanol
6	Intratracheally infected animals gavaged with 5.0% ethanol
7	Uninfected animals gavaged with 275 mg/kg LA
8	Intratracheally infected animals gavaged with 275 mg/kg LA

**Figure 5:**
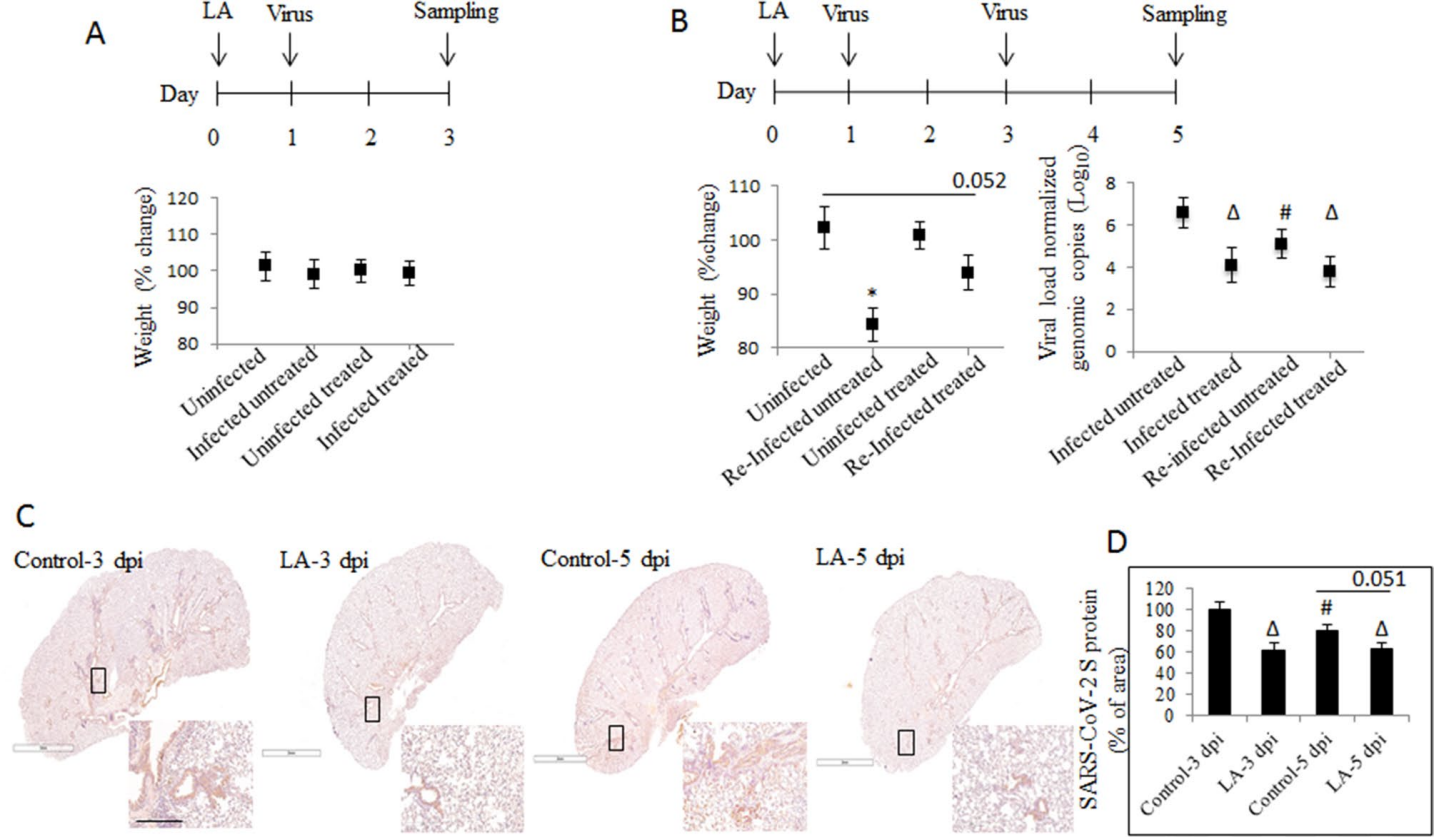
Effect of oral administration of LA against viral replication in vivo. (**A**) Effect of three-day oral administration of LA in K18-hACE2 C57BL/6 J mice with no observed weight change. (**B**) Effect of six-day oral administration of LA in K18-hACE2 C57BL/6 J mice and mid-term re-infected with observed weight change started on day four. The viral burden in lung tissue was determined by RT-qPCR and is represented as VSV L gene copies normalized to mouse Pol2Ra. (**C**) SARS-CoV-2 spike protein detection with immunochemical staining in representative lung tissue sections. (**D**) Quantification of SARS-CoV-2 spike protein performed as described in Material and Method section. Data are presented as mean + / − SD; uninfected untreated animal n = 3, uninfected treated animal n = 3, infected untreated animal n = 8, infected treated animal n = 8, re-infected untreated animal n = 8, re-infected treated animal n = 8, scale bar = 3 mm and 200 μm; LA – linoleic acid; # *p* ≤ 0.05, ∆ *p* ≤ 0.01, * *p* ≤ 0.001, dpi- days post-infection.

Oral LA gavage was carried out in two fashions (Fig. 5). During the first 3 days of infection with rVSVΔG-SARS-CoV-2-S-D614Gd21-NLucP the clinical signs such as respiratory distress, weight loss, apathy, etc., were not observed. Weight loss, compared with control, was manifested only in the infected animals starting on the fourth day from the first infection in a subgroup of animals that were re-infected on the third day (Fig. 5A,B). The viral burden measured in the LA-gavaged groups was also significantly lower in the infected and treated animals compared with infected and not treated controls, with a 1.9 log reduction in viral load in the infected and LA-treated group, and 2.0 log in the re-infected and LA- treated group (Fig. 5B). Interestingly, viral load in the control single-infected animals was significantly 1.1 log higher compared with mice in the control re-infected group. Analysis of spike protein expression in the lung tissues by IHC supported these observations by showing its rather clustered tissue distribution in contrast to more scattered distribution in the lungs of infected and re-infected untreated animals. We noticed that the presence of viral particles and spike protein in the re-infected animals only was significantly lower compared with once- infected animals (Fig. 5C). Quantification of the SARS-CoV-2 spike protein (Fig. 5D) revealed that its presence in the lung of LA-treated and infected and LA-treated and re-infected animals was 38.2% and 37.1% lower compared with a control (infected animals only), respectively. In LA-treated and re-infected animals the spike protein was lower by 17.7% compared with the respective control (i.e., re-infected but not LA-treated).

**Figure 6:**
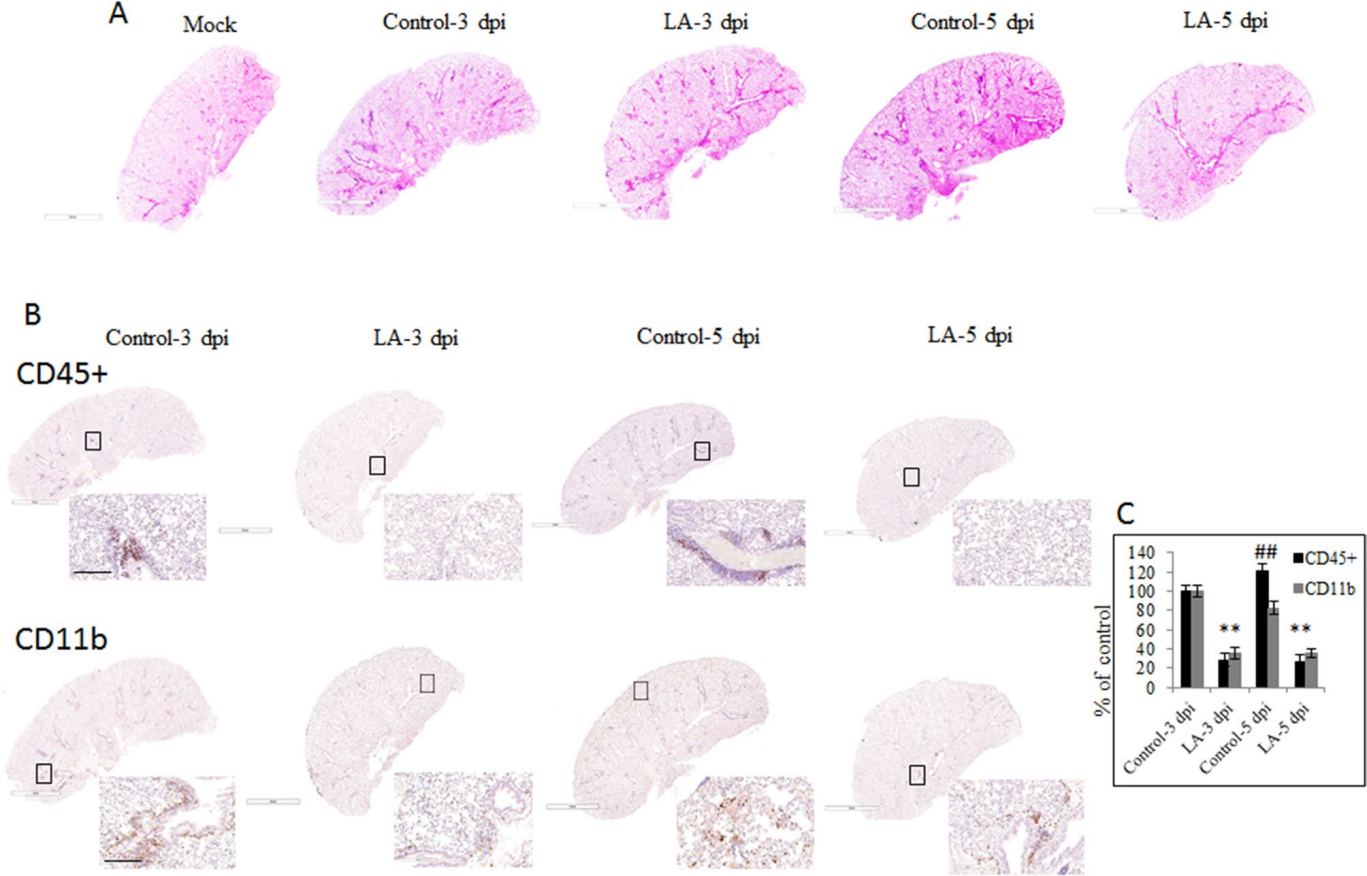
Inflammatory status after mid-term oral administration of linoleic acid in vivo. (**A**) Effect of LA orally gavaged for six days in K18-hACE2 C57BL/6 J mice infected and re-infected with viral particles on lung tissue stained with H&E. (**B**) Effect of LA orally gavaged for six days in K18-hACE2 C57BL/6 J mice and re-infected on lung tissue stained with CD45 + antibody and CD11b antibody. (**C**) Quantification of CD45 + and CD11b antibodies as described in Material and Method section. Data are presented as mean + / − SD; uninfected untreated animal n = 3, uninfected treated animal n = 3, infected untreated animal n = 8, infected treated animal n = 8, re-infected untreated animal n = 8, re-infected treated animal n = 8, scale bar = 3 mm and 200 μm; # *p* ≤ 0.05, ∆ *p* ≤ 0.01, * *p* ≤ 0.001, dpi- days post-infection.

Lung tissues were also subjected to standard histo- and immunochemical analysis by hematoxylin and eosin staining as well as CD45 + and CD11b staining. The analysis of stained slides revealed that the lungs sections from all LA-treated and either once-infected or re-infected animals had less inflammation compared to the corresponding control animals. As such, the LA-treated mice showed a lesser alveolar and peribronchiolar inflammatory representation, compared with re-infected and once-infected untreated mice. All control mice developed pulmonary pathology that could be described as the presence of perivascular leucocytes, hyaline membrane, edema, and infiltrated myeloid cells (Fig. 6A). We observed a 18.9% increase in the presence of CD45 + cells, but a 17.4% decrease of CD11b cells in the control animals re-infected but not treated compared with once-infected control animals. The LA treatment significantly decreased the percentage of CD45 + immune cells in the lungs by about 71.7% (LA-3 dpi) and 72.3% (LA-5 dpi) respectively, compared with infected-only animals (Fig. 6B upper panel, C). The recruitment of CD11b myeloid cells into the lungs of infected mice was about 64.4% (LA-3 dpi) and 64.9% (LA-5 dpi) lower respectively, compared with once infected-only animals (Fig. 6B lower panel, C). We noticed an 18.1% decrease in the presence of CD11b cells in the not-treated and re-infected animals compared with not-treated and only-once-infected animals.

## Discussion

LA (cis, cis-9,12-octadecadienoic acid) is one of the PUFAs commonly found in the human diet. It is an energy source and the origin of myriad metabolites tangled to cellular membrane and various derivatives involved in cell signaling (i.e., 13-hydroxy or 13-hydroperoxy octadecadienoic acid). Concerns that LA can contribute to cardiovascular and inflammatory dysfunctions have not thus far found support from epidemiological and clinical studies^21–29^. Dietary LA is absorbed by enterocytes in the small intestine and incorporated in chylomicrons, which then enter the circulation, delivering LA molecules to hepatic and non-hepatic tissues according to their needs. Emken et al.^32^ have demonstrated that up to 85% of dietary LA may be absorbed by humans. However, no maximum limits for LA have been established thus far, owing to a lack of information on defined intake doses that could trigger any adverse effects^21,23,32^.

The mixed outcome of vaccination against SARS-CoV-2 has refocused the overall goals towards finding potential therapeutic or prophylactic agents, either novel or repurposed. Currently available nucleotide analogs such as remdesivir are in the scope of interest. However, recently published data prompt a caution, owing to the newly emerged remdesivir-resistant mutation E802D in the nsp12. Published clinical evidence indicates that, in an immunocompromised patient who developed a protracted course of SARS-CoV-2 infection, the remdesivir therapy alleviated symptoms but further recovery was complicated by recrudescence of high-grade viral shedding^33^.

Here, we tested the efficacy of 15 FAs, lipid-soluble vitamins such as A, E, and D, and cholesterol, in inhibiting the activity of SARS-CoV-2 RdRp. Preliminary screening allowed for selecting four PUFAs: LA, ALA, EPA, and AA. Interestingly, DHA did not display RdRp inhibition capability. A poor conformational fit of DHA chemical structure to the RdRp is the most likely cause of this. Further experiments testing dose-dependent inhibitory effects have narrowed this selection to LA as a source of the most promising compounds. The LA efficacy was also reproduced in an assay where the overexpressed RdRp was used, although the inhibitory effect was noticed at lower concentrations of LA, i.e., ≤ 25 μg/ml. Experiment, in which mixture of nsp12/nsp7/nsp8 was used provided similar inhibitory response to the one provided by the manufacturer of the kit. This could possibly be attributable to more favorable conformation of the recombinant proteins over the one that was overexpressed in monkey cells.

As Toelzer et al.^34^ showed, there is a binding pocket in RBD-SARS-CoV-2 spike protein for LA, which makes it capable of interfering with viral attachment and affecting the first step of infection. Our earlier published results confirmed that LA inhibits binding of SARS-CoV-2 pseudo-virions to hACE2. However, the binding-inhibitory effects were achieved at higher concentrations than the ones required for inhibition of RdRp activity. As such, in vitro inhibition of SARS-CoV-2 attachment to hACE2 was noticed at LA concentrations of 80 μg/ml and above, whereas inhibition of SARS-CoV-2 RdRp activity, in the study presented here, was observed at and below 75 μg/ml^35^.

Molecular docking revealed that LA binds to the cavity formed by the RNA double helix and protein. It forms hydrophobic interactions with multiple residues, and at the same time forms electrostatic interactions including hydrogen bonding with Lys593 and Asp865. Experimental data from SPR corroborated the computational modeling with calculated K_D_ = 9.3 × 10^7^. In order to ensure that observed binding results, substantiated by a computationally aided model, could be confirmed at the cellular level, but being limited by BSL requirements, we applied three experimental patterns of MRC-5 cells treatment with LA infected with HCoV-OC43 virus (also human RNA type virus that contains a positive, single-stranded RNA) as a substitute for SARS-CoV-2. We observed that either pre-treatment, simultaneous treatment or post-treatment of cells with 10–75 µg/ml LA resulted in a significant, dose-dependent reduction of viral particles after 24 h. Also, the highest concentration of LA used in this study (i.e., 75 µg/ml) significantly reduced the viral genomic copies of HCoV-OC43 depending on its initial application. Importantly, we did not notice that LA (up to 75 µg/ml after 24 h of treatment) affects ACE2 expression at protein level. Since the RdRp structure is rather conservative among RNA viruses, it is plausible that LA could be effective in blocking replication more than just coronavirus strains as well. Another factor to consider is LA’s metabolism in host cells, which could reduce its available amount. This might be reflected in a higher concentration of LA required for RdRp inhibition in the cells compared with the concentrations used in cell-free assays. In addition, it is also possible that LA may incorporate into cellular membranes and/or, in the case of the whole organism, in adipocytes. Taking into account that LA is an ampholytic and membrane-penetrating compound, alterations of the cell membrane properties of either host or capsules of virions, affecting the lipid structure, and as such influencing their fluidity, needs to be acknowledged as well.

To further confirm the inhibitory effect of LA against RdRp, we evaluated the effects of LA administration in an animal model. We used C57BL/6 J mice expressing human ACE2 receptor. Our experiment was limited by the use of HCoV-OC43 and rVSVΔG-SARS-CoV-2-S-D614Gd21-NLucP virions that are deprived of specific infectivity. rVSVΔG-SARS-CoV-2-S-D614Gd21-NLucP virions, however, are replication-competent and infectious, thus suitable for BSL2 + laboratories^36^. We chose to apply non-surgical intratracheal and conventional oral administration of LA. In intratracheal treatment, a short-term viral exposure was significantly reduced by LA. Our results showed effects either similar to or greater than the ones reported for remdesivir in the same mouse model^37^. One consistent outcome observed in both in vitro and in vivo studies was that simultaneous and post-treatment with LA seem to be more effective in decreasing viral load than the pre-treatment with LA. That would accord with our above-mentioned supposition about the influence of LA metabolism and membrane-penetrating competence properties. In both short-term and mid-term oral application, again we observed that administration of LA has a significant antiviral effect as well. This could result in reduced inflammation in lung tissue of infected and LA-treated animals compared with their respective controls, where inflammation occurred. Since inflammation is the leading factor in SARS-CoV-2-triggered lung injury, our findings are of value, and they also clarify still-lingering controversy around LA being considered as a pro-inflammatory compound. We found it interesting that there were fewer viral particles detected in the lung tissue of re-infected animals compared with once-infected animals. This was accompanied by decreased inflammatory infiltration. However, there was an increase in the overall presence of CD45 + cells in re-infected not-treated animals compared with control once-infected animals only. This could be associated with adaptive/ humoral responses facilitated by the spike protein. A similar phenomenon of decreased viral load and peribronchial and perivascular inflammatory infiltration was reported by Jing et al*.*, who used the original SARS-CoV-2 virus at similar 7 × 10^5^ infectious dose^38^. Overall, our in vivo results further confirm a significantly reduced viral replication and viral-triggered inflammation in the lungs of infected and LA-treated mice, as well as a decreased presence of CD45 + cells and infiltration with CD11b (pan-myeloid cells) that were acknowledged as initiators and maintaining factors of lung inflammatory process associated with SARS-CoV-2 infection^38–42^. Based on our results, we can also say that the engineered SARS-CoV-2 virus used by us can be suitable for in vivo experiments to study pulmonary pathologies, especially the aspects of inflammatory responses.

As a note, LA encompasses approximately 20% of all the FAs in human plasma and 9% in cellular membranes of erythrocytes^43^. Abdelmagid et al*.* reported that plasma concentrations of circulating LA accounts in the range of 0.2 to 5.0 mM^44^. Vermunt et al*.* observed that after intake of a single dose of 45 mg of ^13^C-LA, the mean peak of absolute amount of ^13^C-LA reached 3.4 ± 0.8 mg after about 17 h, which accounts for 7.6% of the total ingested dose^45^. This corresponds to mean concentration in plasma total lipids of 953 ± 52 mg/l (i.e., 32.54 ± 1.12% of total FAs) and mean plasma concentrations (Cmax) of 9.3 ± 1.4 mg/l (i.e., 0.31 ± 0.02% of total FAs). In that study, about 21% of the ingested ^13^C-LA was recovered in breath, while the peak amount of ^13^C-LA in plasma was about 8%. Peak of ^13^C-LA in breath was obtained 3–5 h after the intake of C^13^labeled LA. This study also indicated that LA began to be directly converted to its metabolites (mainly ALA), reaching their peak at 25 h, and to diminish after 168 h. Demmelmair et al*.* observed that ^13^C-LA detected in the milk of lactating women reached its peak at 12 h after ingestion of 1 mg ^13^C-LA/kg body weight, with a similar time to peak (Tmax) in the breath reported by Vermunt et al*.*, and the total recovery of 18–24%^46^. By comparison, consumption of 140 mg of LA by rats resulted in a Cmax of 102 ± 11 nmol/ml, at Tmax 6.45 ± 0.05h^47^. By our assessments, LA inhibited in vitro SARS-CoV-2 replication in a dose-dependent fashion when applied at the range between 17.8 and 267.4 µM. In our in vivo study, a single dose of 75 μg of LA administrated intratracheally, and a 5.5–6.88 mg daily dose administrated orally, resulted in lowering the viral load in the lung tissue and at the same time hindering the inflammatory burden. However, since our studies were limited to using the engineered SARS-CoV-2 virus, in order to comply with the safety issues, the use of the original SARS-CoV-2 virus instead is warranted.

In conclusion, PUFAs, in particular LA, manifested a direct inhibitory effect on RdRp activity, and repressed viral replication. The results presented here lay the foundation for further studies to expand our understanding of FAs’ efficacy against SARS-CoV-2.

## Material and methods

### Cells, viruses, test compounds, antibodies and inhibitors

MRC-5 cell line, human coronavirus OC43 (HCoV-O43) and human coronavirus 229E (HCoV-229E) were obtained from ATCC (American Type Culture Collection) (Manassas, VA) and maintained in Modified Eagle’s Medium containing 10% fetal bovine serum. Replication-competent SARS-CoV-2 as rVSVΔG-SARS-CoV-2-S-D614GΔ21-NLucP was purchased from Kerafast (Boston, MA). rVSVΔG-SARS-CoV-2-S-D614Gd21-NLucP is a recombinant vesicular stomatitis virus, in which the native glycoprotein has been replaced with the SARS-CoV-2 spike protein lacking the last 21 residues of the cytoplasmic tail, and contains the D614G amino acid change (S_Met1_D614GΔ21), thus the virus is capable of interacting with and entering cells through the SARS-CoV-2 spike, but once fusion occurs, it replicates using the vesicular stomatitis virus (VSV) machinery. SARS-CoV-2 RdRp complex (i.e., nsp12/nsp7/nsp8) was from BPS Bioscience (San Diego, CA). All FAs (except EPA and DHA) and vitamin A (all-trans-retinol) were obtained from Cayman Chemical Company (Ann Arbor, MI), and other lipid-soluble vitamins, and cholesterol were purchased from Sigma (St. Louis, MO). HCoV-OC43 anti-spike antibody was from CusaBio Technology LLC (Houston, TX), SARS-CoV-2 anti-spike antibody was from GeneTex, Irvine, CA, whereas anti-ACE2 and anti-β-actin antibody were from Santa Cruz Biotechnology (Santa Cruz, CA). Anti-CD45 + and anti-CD11b antibodies were from Cell Signaling (Danvers, MA).

### SARS-CoV-2 RdRp expression

RdRp expression was performed according to published methodology (16). Target DNA sequence of RdRp (sequence of SARS-CoV-2 strain Wuhan-Hu-1) was taken from Genbank accession YP_009725307. The SARS-CoV-2 RdRp gene (as nsp 12) was codon-optimized for expression in insect cells, then synthetized and sub-cloned into a target vector for insect cell expression (i.e., construct pFAStBac1-RdRp-His-Strep) (GenScript, Piscataway, NJ). DH10Bac strain was then used for recombinant bacmid (rBacmid) generation. The positive rBacmid- containing the RdRp gene was selected and confirmed by sequencing. Sf9 cells were grown in Sf-900 II SFM Expression Medium (Life Technologies, Carlsbad, CA). The cells were maintained in a 27 °C flask in an orbital shaker. One day before transfection, the cells were seeded in a 6-well plate. On the day of transfection, DNA and transfection reagent (Promega, Madison, WI) were mixed together at the optimal ratio and then added into the plate with the cells ready for transfection. Cells were incubated in Sf-900 SFM for 5–7 days at 27 °C before harvest. The supernatant was collected after centrifugation and designated as P1 viral stock. Viral stock designated as P2 was amplified for later infection. The expression was analyzed by western blot. The 1 L of SF9 cell culture was infected by P2 viral stock. Cells were incubated in Sf-900 II for 3 days at 27 °C before harvest. The expression was again analyzed by western blot. Cell pellet was harvested, lysed in cell buffer [300 mM NaCl, 50 mM Na-HEPES pH 7.4, 10% (v/v) glycerol, 30 mM imidazole pH 8.0, 3.0 mM MgCl_2_, 5 mM β-mercaptoethanol, 0.284 μg/ml leupeptin, 1.37 μg/ml pepstatin, 0.17 mg/ml PMSF, and 0.33 mg/ml benzamidine], and sonicated. The cell lysate (i.e., supernatant) was incubated with Ni–NTA to capture the target protein and then further purified by Superdex 200 16/600GL (GE Healthcare, Chicago, IL). Fractions with the higher purity were pooled, followed by 0.22 μm filter sterilization, and stored in − 80 °C. Proteins were analyzed by SDS-PAGE and western blot. Concentration was determined by Bradford protein assay. Synthesis of RdRp gene and sequencing of positive rBacmid was performed by GenScript (Piscataway, NJ).

### Surface plasmon resonance (SPR) binding assay

SPR binding assay, which measures binding affinity K_D_ as well kinetic parameters ka and kd of human RdRp protein, with LA as the ligand, was utilized, where RdRp protein was printed onto the chip and the analyte was LA at different concentrations (Creative Biostructure, Shirley, NY). The bare gold-coated (thickness 47 nm) PlexArray Nanocapture Sensor Chip (Plexera Bioscience, Seattle, WA) was prewashed with 10 × PBST (PBS + Tween-20) for 10 min, 1 × PBST for 10 min, and deionized water twice for 10 min, before being dried under a stream of nitrogen prior to use. Various concentrations of human RdRp protein dissolved in water were manually printed onto the chip with Biodo bio-printing at 40% humidity via biotin-avidin conjugation. Each concentration was printed in replicate, and each spot contained 0.2 µl of sample solution. The chip was incubated in 80% humidity overnight at 4 °C, and rinsed with 10 × PBST for 10 min, 1 × PBST for 10 min, and deionized water twice for 10 min. The chip was then blocked with 5% (w/v) non-fat milk in water overnight, and washed with 10 × PBST for 10 min, 1 × PBST for 10 min, and deionized water twice for 10 min before being dried under a stream of nitrogen prior to use. SPR measurements were performed with PlexAray HT (Plexera Bioscience, Seattle, WA). Collimated light (660 nm) was passed through the coupling prism reflecting off the SPR-active gold surface, and was received by the CCD camera. Buffers and samples were injected by a non-pulsatile piston pump into the 30 µl flowcell that was mounted on the coupling prism. Each measurement cycle contained four steps: washing with PBST running buffer at a constant rate of 2.0 µl/s to obtain a stable baseline, sample injection at 5 µl/s for binding, surface washing with PBST at 2 µl/s for 300 s, and regeneration with 0.5% (v/v H_3_PO_4_ at 2 µl/s for 300 s. All the measurements were performed at 25 °C. The signal changes after binding and washing (in AU) were recorded as the assay value. Kinetics fitting and analysis-selected protein-grafted regions in the SPR images were analyzed, and the average reflectivity variations of the chosen areas were plotted as a function of time. After data collection with SPR and kinetics fitting and analysis, the K_D_, ka and kd were calculated. Real-time binding signals were recorded and analyzed by Data Analysis Module (DAM, Plexera Bioscience, Seattle, WA). Kinetic analysis was performed using BIA evaluation 4.1 software (Biacore, Inc.).

### Molecular docking

The structure of PDB code 7c2k was downloaded from the PDB library (www.rcsb.org). It is a complex structure of SARS-CoV-2 RNA polymerase with double-strand RNA fragments (Creative Biostructure, Shirley, NY). The three-dimensional structure of the LA molecule in the sdf format was downloaded from pubchem (https://pubchem.ncbi.nlm.nih.gov/compound/Linoleic-acid), and OpenBabel (30) was used to convert it into the mol2 file for further processing. In this study, AutoDock Vina (31) was selected as the molecular docking tool. MGLTools 1.5.6 was used to read in 7c2k, and performed hydrogenation and gave Kollman charge to generate the protein.pdbqt file. With reference to the compound structure 7bv2 of RdRp and Radixivir, the center coordinates of the docking box x, y, z were defined as 133.1, 139.1, 141.1, based on the binding position of Radixivir, and the box size was set to 30 × 30 × 30 Å, so it could contain the entire pocket area. The ligand_prepare.py script in the molecular docking package was used to deal with the mol2 file of the ligand linoleic acid. The flexible bond was set by default, and Gasteiger charge was added to generate the ligand pdbqt file. The exhaustiveness value of the search parameter was set to 10 and defined to output the top 9 ranking conformations according to docking scores. The default values were selected for the rest of the parameters.

### SARS-CoV-2 RdRp activity assays

*Cell-free assay with recombinant RdRp:* RdRp activity was evaluated using a SARS-CoV-2 RNA Polymerase Assay Kit (ProFoldin, Hudson, MA) according to the manufacturer’s protocol. Briefly, 0.5 µl of 50 × SARS-CoV-2 recombinant RdRp was incubated with 2.5 µl of 50 × buffer, 20 µl of water, and 1.0 µl of FA at 0, 10, 25, 50, and 75 μg/ml concentrations (i.e., 0, 35.7, 89.2, 178.3, and 267.4 μM) for 15 min at RT, followed by the addition of master mix containing 0.5 µl of 50 × NTPs and 0.5 µl of 50 × template (as a single-stranded polyribonucleotide). The reaction (25 µl) was incubated for 2 h at 34 °C and then stopped by the addition of 65 µl of 10 × fluorescence dye, and the fluorescence signal was recorded in 10 min at extension/emission = 488/535 nm using a fluorescence spectrometer (Tecan, Group Ltd., Switzerland). Results are expressed as a % of control without test compound (mean + / − SD, n = 6).

*Cell-free assay with recombinant RdRp (complex of nsp12/nsp7/nsp8):* RdRp activity was evaluated using a RNA Polymerase Assay Kit (ProFoldin, Hudson, MA) according to the manufacturer’s protocol. Briefly, 1 µl (25 ng) SARS-CoV-2 recombinant RdRp complex was incubated with 2.5 µl of 50 × buffer, 20 µl of water, and 1.0 µl of FA at 0, 10, 25, 50, and 75 μg/ml concentrations (i.e., 0, 35.7, 89.2, 178.3, and 267.4 μM) for 15 min at RT, followed by the addition of master mix containing 0.5 µl of 50 × NTPs and 0.5 µl of 50 × template (as a single-stranded polyribonucleotide). The reaction (25 µl) was incubated for 2 h at 34 °C and then stopped by the addition of 65 µl of 10 × fluorescence dye, and the fluorescence signal was recorded in 10 min at extension/emission = 488/535 nm using a fluorescence spectrometer (Tecan, Group Ltd., Switzerland). Results are expressed as a % of control without test compound (mean + / − SD, n = 6).

*Cell-free assay with overexpressed RdRp:* To determine the inhibitory effect of LA on SARS-CoV-2 RdRp overexpressed in Vero cells, 20 µl of cell lysate/20 µg protein was incubated with 2.5 µl of 50 × buffer and 1.0 µl of LA at 0, 10, 25, 50, and 75 μg/ml concentrations (i.e., 0, 35.7, 89.2, 178.3, and 267.4 μM), respectively, for 15 min at RT, followed by the addition of master mix containing 0.5 µl of 50 × NTPs and 0.5 µl of 50 × template (as a single-stranded polyribonucleotide). The reaction (25 µl) was incubated for 2 h at 34 °C and then stopped by addition of 65 µl of 10 × fluorescence dye, and the fluorescence signal was recorded after 10 min at extension/emission = 488/535 nm using a fluorescence spectrometer (Tecan, Group Ltd., Switzerland). Results are expressed as a % of control without test compound (mean + / − SD, n = 6).

To transduce cells with eGFP-luciferase-SARS-CoV-2 RdRp lentivirus (GenScript, Piscataway, NJ), Vero cells seeded into a 6-well plate in the presence of complete growth medium were treated with 8 µl/ml polybrene (Sigma, St. Louis, MO) for 30 min, followed by the addition of eGFP-luciferase-SARS-CoV-2 RdRp lentivirus (GenScript, Piscataway, NJ) at a multiplicity of infection (MOI) of 5 (our previous preliminary results showed an almost 100% transduction rate can be achieved with this MOI), and spin-inoculation at 1000 × g for 1 h. After 24 h at 37 °C incubation, cells were fed with fresh complete growth medium. After 48 h post-inoculation, cells were detached with 1.0 mM EDTA, washed twice with 1 × PBS (phosphate-buffered saline), and disrupted with a Dounce tissue homogenizer and sonication. Efficacy of transduction was validated by western blot with anti-RdRp antibody at dilution 1:1000 (Kerafast, Boston, MA).

### In vitro replication assays

In vitro* pre-treatment study*: MRC-5 cells were seeded in a 6-well plate at 1.0 × 10^6^. After attachment, the growth media were replaced with maintenance media containing 0, 10, 25, 50, and 75 µg/ml of LA, or DMSO-only control. Treated plates were incubated in a 33 °C 5% CO_2_ incubator for 2 h. After pre-treatment, HCoV-O43 particles were added for 3 h to all wells, yielding a MOI of 0.1. The growth medium was then replaced with a fresh one and the infection was allowed to proceed for 24 h at 33 °C in 5% CO_2_. Next, conditioning media were collected and viral load in conditioning medium was determined by RT-qPCR. Results are expressed as a log_10_ genomic copies/ml compared to LA-free control (mean + / − SD, n = 6).

In vitro* treatment study*: MRC-5 cells were seeded in a 6-well plate at 1.0 × 10^6^. After cell attachment, the growth media were replaced with maintenance media containing 0, 10, 25, 50, and 75 µg/ml of LA, or DMSO-only control, and either HCoV-O43 particles were added to all wells, yielding an MOI of 0.1. The incubation was allowed to proceed for 3 h at 33 °C in 5% CO_2._ The growth medium was then replaced with a fresh one and the infection was allowed to proceed further for 24 h at 33 °C and 5% CO_2_. Next, conditioning media were collected and viral load in conditioning medium was determined by RT-qPCR. Results are expressed as a log_10_ genomic copies/ml compared to LA-free control (mean + / − SD, n = 6).

In vitro* post-treatment study*: MRC-5 cells were seeded in a 6-well plate at 1.0 × 10^6^. After cell attachment, the growth media were replaced with maintenance media containing HCoV-O43 added to all wells for 3 h, yielding an MOI of 0.1, after which 0.1. 5, 10, 25, 50, and 75 µg/ml of LA, or DMSO-only control was added. After the next 2 h, the growth medium was replaced with a fresh one and the infection was allowed to proceed for 24 h at 33 °C and 5% CO_2_. Next, conditioning media were collected and viral load in conditioning medium was determined by RT-qPCR. Results are expressed as a log_10_ genomic copies/ml compared to LA-free control (mean + / − SD, n = 6).

In vitro* viral dose-addition study*: MRC-5 cells were seeded in a 6-well plate at 1.0 × 10^6^. After cell attachment, the growth media were replaced with maintenance media containing HCoV-O43 added to all wells for 3 h, yielding an MOI of 0.01, 1.0, and 10.0 concurrently with single 75 µg/ml of LA concentration, or DMSO-only control was added. After the next 2 h, the growth medium was replaced with a fresh one and the infection was allowed to proceed for 24 h at 33 °C and 5% CO_2_. Next, conditioning media were collected and viral load in conditioning medium was determined by RT-qPCR. Results are expressed as a log_10_ genomic copies/ml compared to LA-free control (mean + / − SD, n = 6).

### Cytotoxic assays

*Cell viability without virus:* To assess cell viability, CellTiter 96^®^AQueous One Solution (Promega, Madison, WI) was used. Briefly, Vero cells were seeded into a 96-well plate at a cell density of 5 × 10^4^ per well and allowed to adhere for 24 h, followed by treatment with serially diluted selected LA for 24 h. Next, 100 μl CellTiter 96^®^AQueous One Solution was added according to the manufacturer’s protocols. The absorbance was measured using a microplate spectrophotometer (Molecular Devices, San Jose, CA). Results are expressed as a % of LA-free control (mean + / − SD, n = 10).

*Cell viability with virus:* To assess cell viability, Vero cells were seeded into a 96-well plate at a cell density of 5 × 10^4^ per well and allowed to adhere for 24 h, followed by treatment with serially diluted selected LA and HCoV-OC43, yielding an MOI of 0.1. The cell viability was assessed using CellTiter 96^®^AQueous One Solution (Promega, Madison, WI) after 24 h, following the manufacturer’s protocols. The absorbance measurements were done using a microplate spectrophotometer (Molecular Devices, San Jose, CA). Results are presented as a % of LA-free control (mean + / − SD, n = 10).

### In vivo study

K18-hACE2 C57BL/6 J 6–8-week-old mice weighing approximately 20–25 g were used in this study (The Jackson Laboratory, Bar Harbor, ME). The mice were kept at an ambient temperature of 21 °C with fat-deprived standard rodent diet (with adjusted calories content) and water provided ad libitum during a light and dark cycle of 12 h. Experimental animal protocol No. 02/B042021 was reviewed by and approved in 2021 by the Animal Care and Use Committee at the Dr. Rath Research Institute. The study is reported in accordance with ARRIVE guidelines as well as all methods were performed in accordance with the relevant guidelines and regulations. Mice were randomly divided into 8 experimental groups as presented in Table 3. Either 1 × 10^5^ tissue infectious doses (TCID50) of HCoV-O43 or 3 × 10^5^ (TCID_50_ of rVSVΔG-SARS-CoV-2-S-D614Gd21-NLucP particles were inoculated intratracheally into the animals from infected groups as well as infected and LA-treated groups. In addition, animals from infected group as well as infected and LA-gavaged group were mid-term intratracheally re-infected with continued oral administration of LA. Oral gavaging began 24 h prior to viral infection, and the weight of gavaged animals was monitored daily. At the end of each study, all animals were sacrificed by overdosing of isoflurane, and the lung samples were collected, immediately snap-frozen in liquid nitrogen, and subjected to RT-qPCR and western blot test. For histology/immunohistochemistry, tissues were fixed in 10% neutral-buffered formalin for 3 days and moved to 70% of ethanol and stored until histology/immunohistochemistry was performed.

### Quantification of RNA

In vitro* study:* HCoV-O43 load in conditioning medium was quantified by extracting RNA using Zymo Quick-RNA Viral 96 Kit (Zymo Research, Irvine, CA) according to the manufacturer’s protocol. Next, TaqMan Advanced Master Mix exploiting one-step RT-qPCR principle was used in triplicates for each sample with TaqMan Gene Expression System (ThermoFisher, Waltham, MA) on BioRad CFX instrument (Hercules, CA), with cycling parameters as: 20 s at 95 °C; and 40 cycles at 95 °C for 3 s and 60 °C for 30 s. Standard curve of viral copy number/CT value was created using serially diluted positive control of know concentrations of HCoV-OC43 RNA (ATCC, Manassas, VA).

In vivo* study:* HCoV-O43 genome copies in lung tissues were quantified by extracting RNA with a Qiagen RNA Plus Isolation kit (Germantown MD), according to the manufacturer’s protocol. 2.0 µg/ml extracted RNA was then taken for cDNA synthesis using a High-Capacity RNA-to-cDNA kit, following the manufacture’s protocol (ThermoFisher, Waltham, MA). Two-step RT-qPCR with TaqMan Advanced Master Mix and TaqMan Gene Expression Assay (ThermoFisher, Waltham, MA) was then executed in triplicates for each sample on BioRad CFX instrument (Hercules, CA), with cycling parameters as: 20 s at 95 °C; and 40 cycles at 95 °C for 3 s and 60 °C for 30 s. Genomic copies per lung tissue were normalized to the relative expression of the mouse RNA Polymerase II gene (Pol2Ra) using TaqMan™ Gene Expression Assays (ThermoFisher, Waltham, MA). rVSVΔG-SARS-CoV-2-S-D614Gd21-NLucP genome copies in lung tissues were quantified by extracting RNA with a Qiagen RNA Plus Isolation kit (Germantown MD), according to the manufacturer’s protocol. 2.0 µg/ml extracted RNA was then taken for cDNA synthesis using a High-Capacity RNA-to-cDNA kit, following the manufacture’s protocol (ThermoFisher, Waltham, MA). Two-step RT-qPCR exploiting the TaqMan^®^ principle was then executed in triplicates for each sample using Vesicular Stomatitis Virus Polymerase (L) Gene^®^ Genesig Advanced Kit (Primerdesign Ltd, Plymouth Meeting, PA) and BioRad CFX instrument (Hercules, CA), with cycling parameters as: 2 min. at 95 °C; and 50 cycles at 95 °C for 10 s and 60 °C for 60 s. Viral copies of SARSCoV-2 were detected using primers for the L region of the VSV genome. Genomic copies per lung tissue were normalized to the relative expression of the mouse RNA Polymerase II gene (Pol2Ra) using TaqMan™ Gene Expression Assays (ThermoFisher, Waltham, MA).

### Immunohistochemistry

Lung tissues were immediately fixed after harvesting, embedded in paraffin, subjected to sectioning (5 μm sections), and stained with hematoxylin and eosin (H&E) as well as with antibodies against SARS-CoV-2 spike protein, CD45+ , and CD11b, respectively. For H&E staining, lung sections were processed following standard histological routine. For indirect immunohistochemistry (IHC), lung sections were also processed following standard IHC routine slides with antigen retrieval (i.e., 15 min heat-induced with EDTA pH 6.0). Primary antibodies were used as follow: anti-spike protein at 1:200, anti-CD45+ at 1:200, and CD11b at 1:200. Taken images were scanned with Aperio AT2 system (Leica, Buffalo Grove, IL). The areas with detected spike protein, CD45+ , and CD11b, were divided by the sum of the areas corresponding to cellular structures counterstained with hematoxylin + anti-spike protein, anti-CD45+ , and anti-CD11b, respectively. Calculated ratios are represented as a % of control. All histology and IHC was performed cryptically coded sections by third party at the Inotiv facility (Boulder, CO).

### Western blot analysis

Cells were treated with indicated concentrations of LA and lysed using lysis buffer [50 mM Tris–HCl (pH = 7.4), 1.0% Triton X-100, 150 mM NaCl, 1.0 mM EDTA, 2.0 mM Na_3_VO_4_, and 1 X complete protease inhibitors (Roche Applied Science, Indianapolis, IN)]. Lung tissue samples were also lysed with the same lysis buffer. The protein concentration was measured by the Dc protein assay (Bio-Rad, Hercules, CA). An 80 µg/well of protein was separated on 8–16% gradient SDS-PAGE gels (i.e., Tris-based electrophoresis using standard Laemmle’s method) and transferred to a PVDF membrane. Proteins were detected either with commercially available anti-spike antibody at 1:500 dilution, anti-ACE2 antibody at 1:500 dilution, and anti-β-actin antibody at 1:2500 dilution as a loading control. WB images were acquired using the Azure cSeries system and auto-exposure settings (Azure Biosystems, CA).

### Statistical analysis

Data for all experiments, unless indicated otherwise, are presented as an average value and standard deviation from at least three independent experiments, each at least in three replicates. Comparison between different samples was done by a two-tailed T-test using the Microsoft Office Excel program. Differences between samples were considered significant at p values less than 0.05.

## Supplementary Information

Supplementary Information.

## Data Availability

All data are contained within the manuscript.
