# Supplementary material for: Linoleic acid binds to SARS-CoV-2 RdRp and represses replication of seasonal human coronavirus OC43
Source: Sci Rep. 2022 Nov 9;12:19114. doi: 10.1038/s41598-022-23880-9 (PMC9645759; doi:10.1038/s41598-022-23880-9)
Supplement: Supplementary file 1 — Supplementary Information. [file 41598_2022_23880_MOESM1_ESM.pptx]

## Slide 1
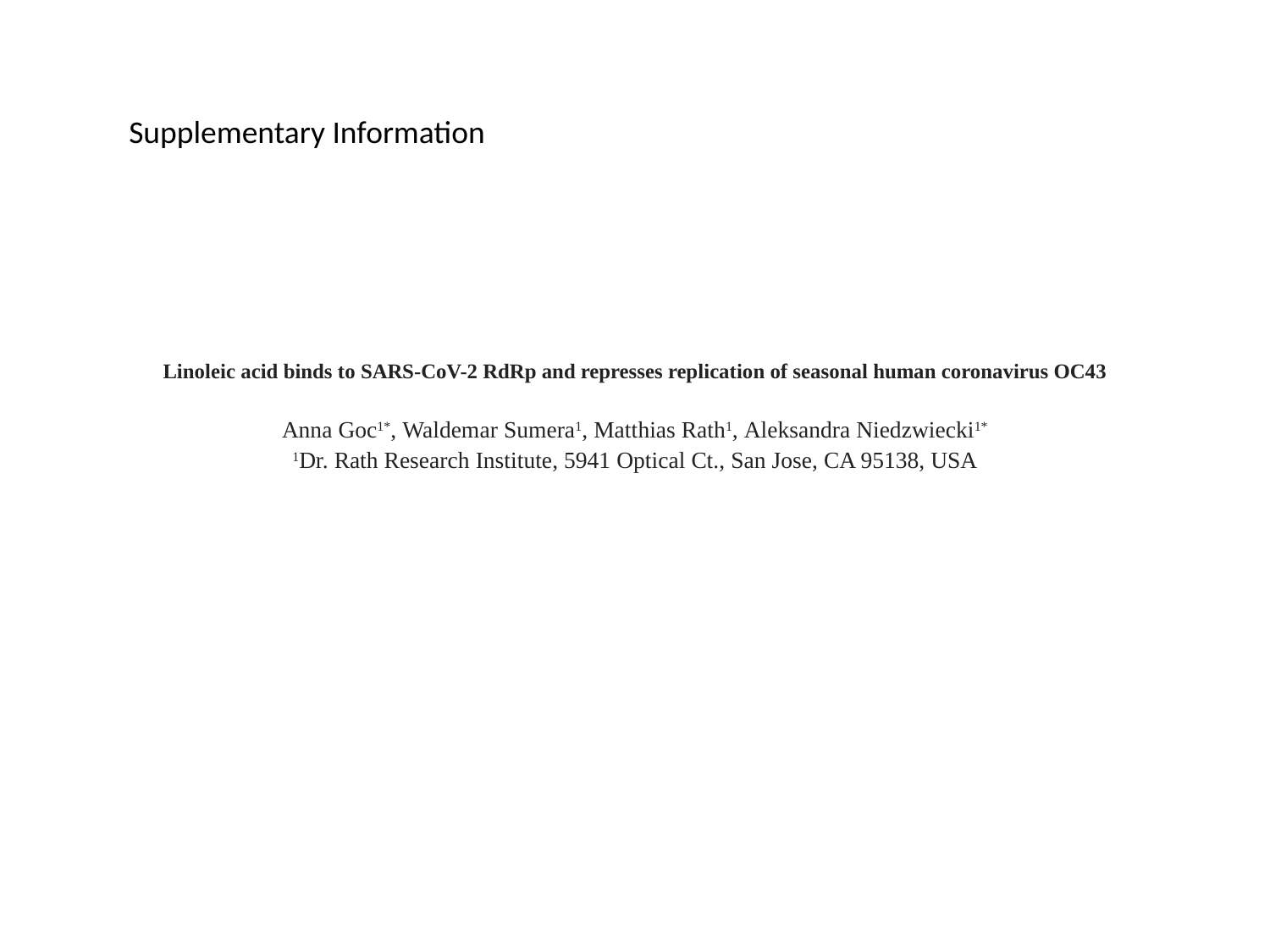

Supplementary Information
# Linoleic acid binds to SARS-CoV-2 RdRp and represses replication of seasonal human coronavirus OC43   Anna Goc1*, Waldemar Sumera1, Matthias Rath1, Aleksandra Niedzwiecki1* 1Dr. Rath Research Institute, 5941 Optical Ct., San Jose, CA 95138, USA

## Slide 2
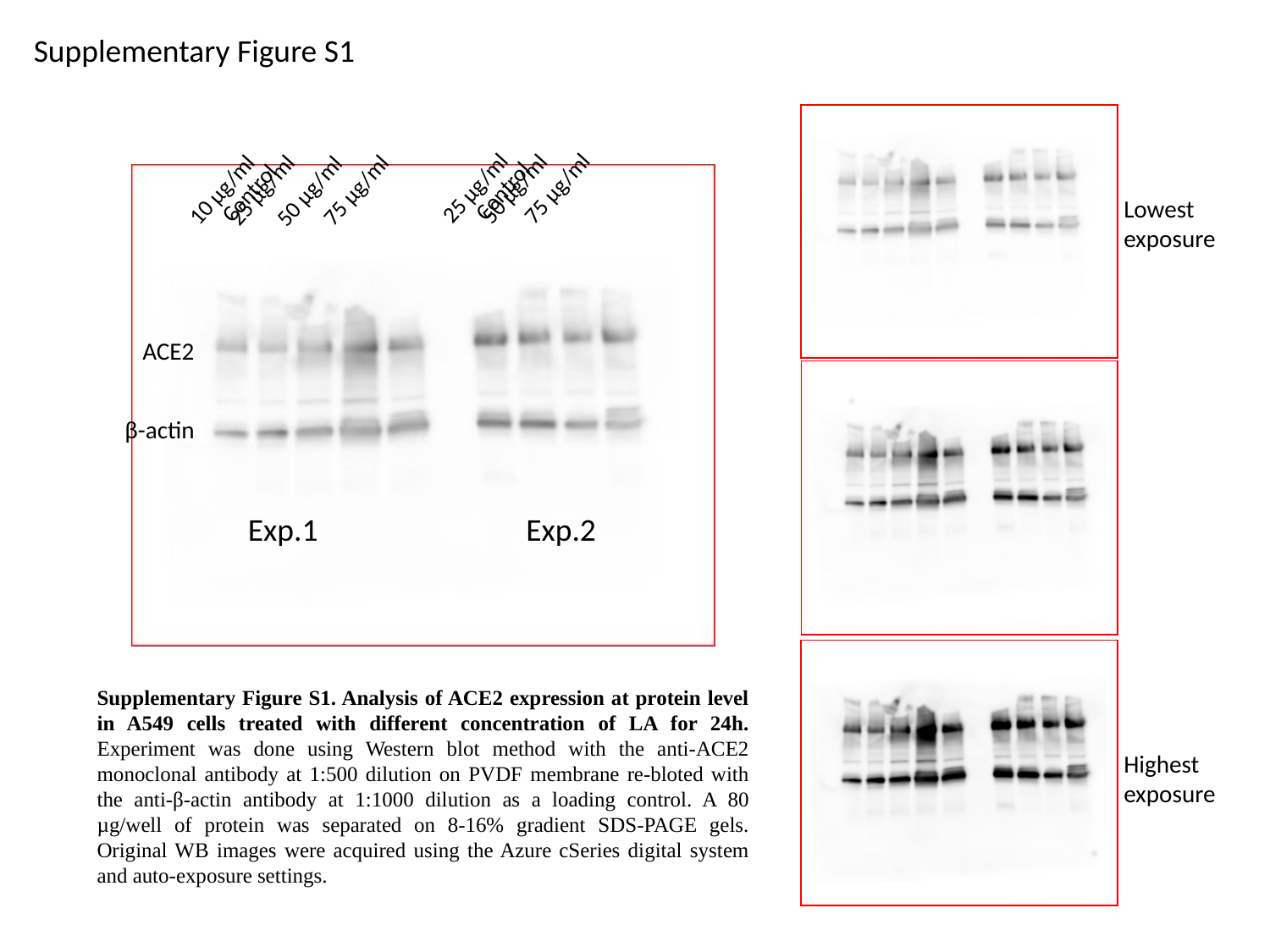

Supplementary Figure S1
25 µg/ml
75 µg/ml
50 µg/ml
10 µg/ml
25 µg/ml
75 µg/ml
50 µg/ml
Control
Control
Lowest exposure
ACE2
β-actin
Exp.1 Exp.2
Supplementary Figure S1. Analysis of ACE2 expression at protein level in A549 cells treated with different concentration of LA for 24h. Experiment was done using Western blot method with the anti-ACE2 monoclonal antibody at 1:500 dilution on PVDF membrane re-bloted with the anti-β-actin antibody at 1:1000 dilution as a loading control. A 80 µg/well of protein was separated on 8-16% gradient SDS-PAGE gels. Original WB images were acquired using the Azure cSeries digital system and auto-exposure settings.
Highest exposure

## Slide 3
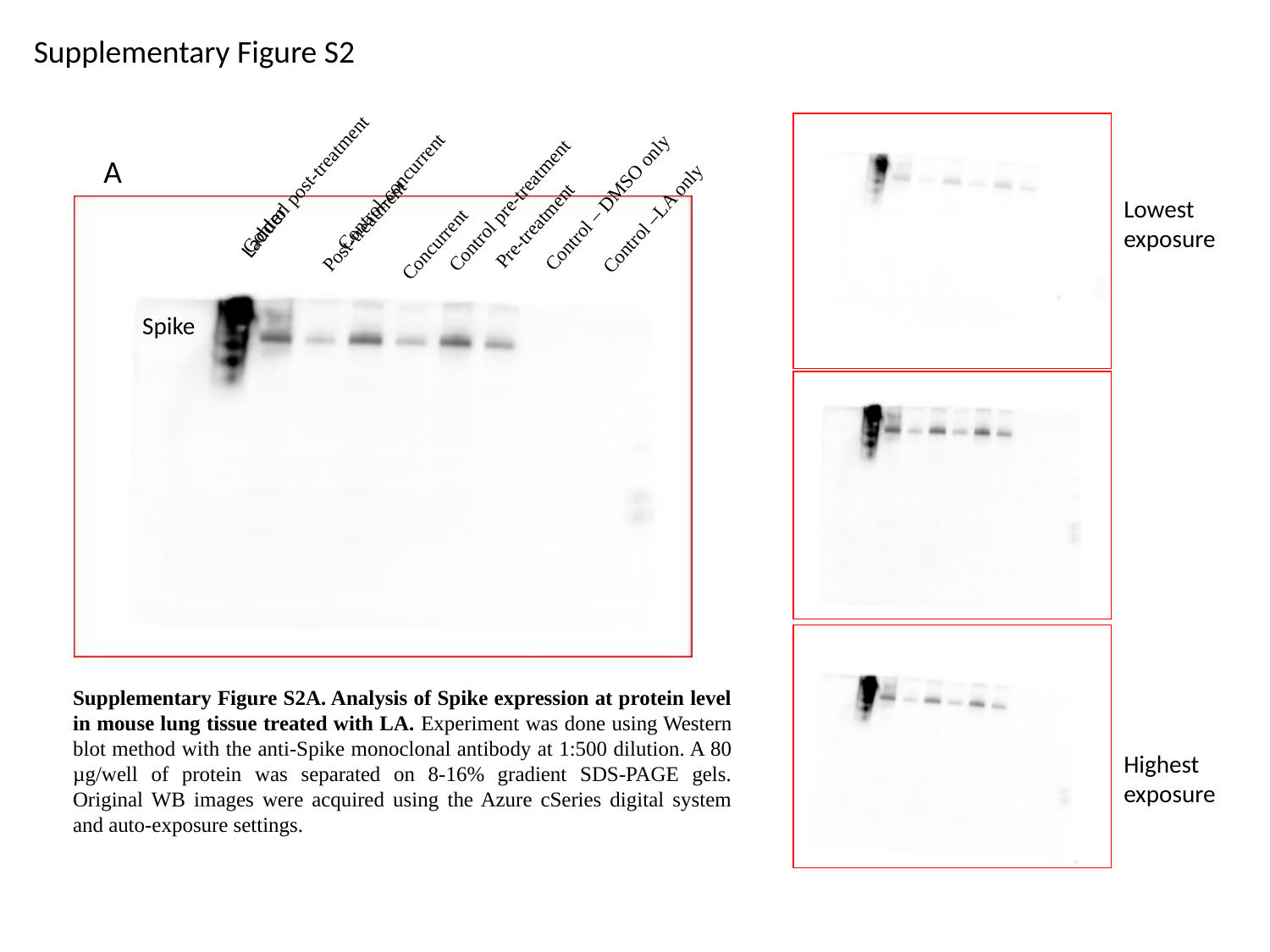

Supplementary Figure S2
A
Control-concurrent
Control post-treatment
Control – DMSO only
Control pre-treatment
Lowest exposure
Control –LA only
Ladder
Pre-treatment
Post-treatment
Concurrent
Spike
Supplementary Figure S2A. Analysis of Spike expression at protein level in mouse lung tissue treated with LA. Experiment was done using Western blot method with the anti-Spike monoclonal antibody at 1:500 dilution. A 80 µg/well of protein was separated on 8-16% gradient SDS-PAGE gels. Original WB images were acquired using the Azure cSeries digital system and auto-exposure settings.
Highest exposure

## Slide 4
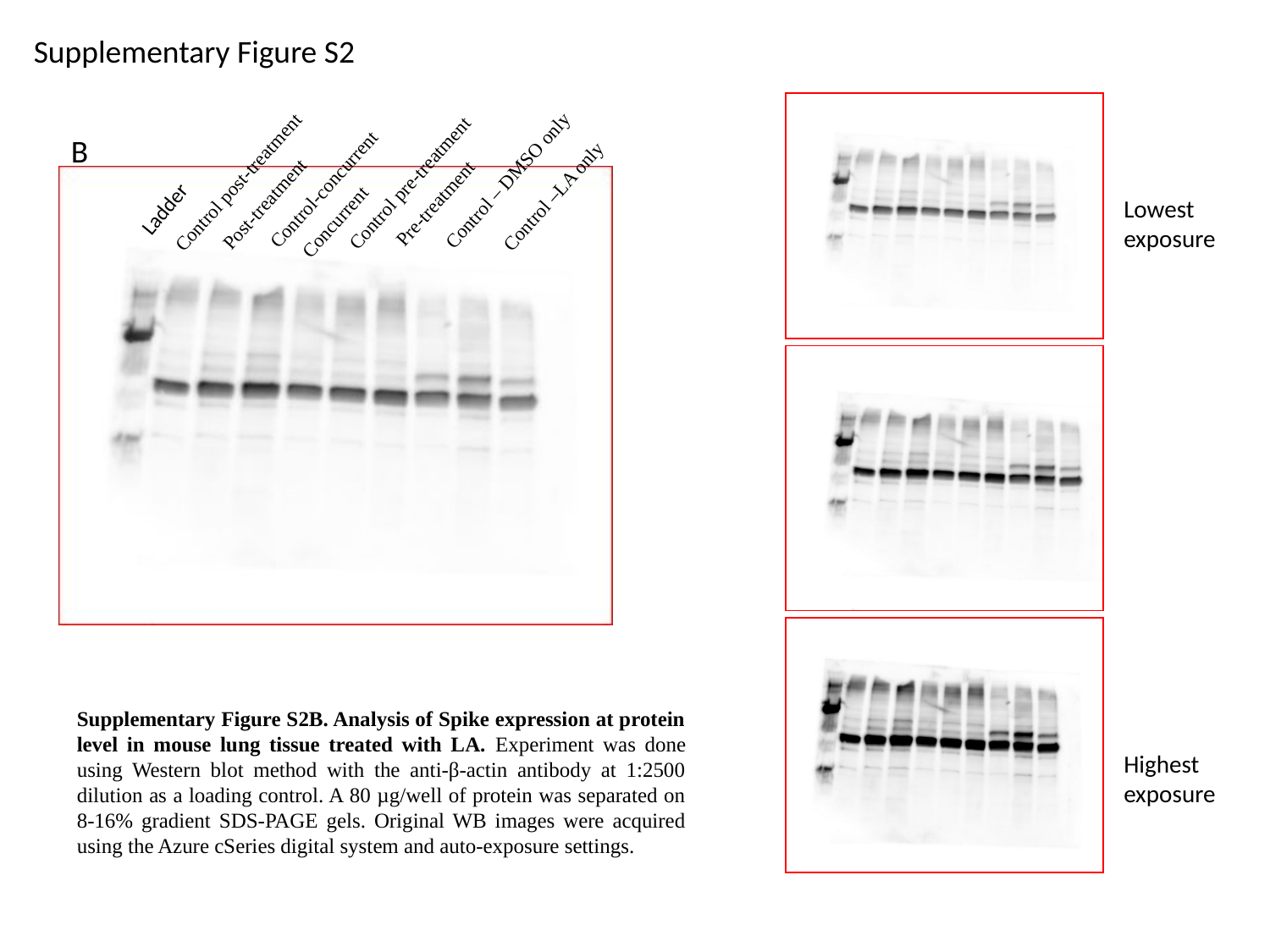

Supplementary Figure S2
B
Control-concurrent
Control – DMSO only
Control post-treatment
Control pre-treatment
Control –LA only
Ladder
Pre-treatment
Post-treatment
Lowest exposure
Concurrent
β-actin
Supplementary Figure S2B. Analysis of Spike expression at protein level in mouse lung tissue treated with LA. Experiment was done using Western blot method with the anti-β-actin antibody at 1:2500 dilution as a loading control. A 80 µg/well of protein was separated on 8-16% gradient SDS-PAGE gels. Original WB images were acquired using the Azure cSeries digital system and auto-exposure settings.
Highest exposure
